# Staff nurses' perceptions of toxic leadership behaviors in nurse managers: a latent profile analysis

**DOI:** 10.3389/fpsyg.2026.1663057

**Published:** 2026-02-18

**Authors:** Xueyu Yan, Xuelian Yan, Li Tan, Hu Jiang

**Affiliations:** The First People's Hospital of Zunyi, The Third Affiliated Hospital of Zunyi Medical University, Zunyi, China

**Keywords:** clinical nurse, cross-sectional study, latent profile analysis, nurse managers, toxic leadership behaviors

## Abstract

**Objectives:**

To identify latent profiles and influencing factors of toxic leadership behaviors of nurse managers experienced by staff nurses.

**Design:**

Cross-sectional study.

**Setting:**

A total of 12 public hospitals in Guiyang and Zunyi city, Guizhou Province, China.

**Methods:**

From May 7, 2024 to December 31, 2024, a total of 900 nurses participated, and 868 valid questionnaires were collected with a validity rate of 96.44%. Data was collected via the Toxic Leadership Behaviors of Nurse Managers scale and a demographic questionnaire. Using latent profile analysis (LPA), distinct profiles of toxic leadership behaviors among nurse managers were identified. Univariate and multiple logistic regression analyses were performed to identify the factors associated with the toxic leadership behavior of nurse managers.

**Results:**

The toxic leadership behaviors suffered by nurses were divided into four profiles: low toxic leadership behavior group (55.07%), moderate toxic leadership behavior group (16.71%), high toxic leadership behavior group (13.36%), and high Intemperate behavior group (14.86%). The results of multiple logistic regression analysis showed that nurses who are male, employed as non-permanent staff, or working in general hospitals are more susceptible to toxic leadership behaviors.

**Conclusions:**

This study used latent profile analysis to identify four distinct subgroups and found that male nurses, non-permanent staff, and nurses in general hospitals are more susceptible to toxic leadership behaviors. These results emphasize the need for developing strategies to address toxic leadership behaviors in order to promote nurses' wellbeing.

## Introduction

As the coordinator of the nursing team, nursing managers should create a favorable professional environment for nurses by making sound management decisions and effectively allocating resources (Alanazi et al., 2023). The effective performance of these important management functions depends on the pattern of leadership behaviors adopted by managers. Leadership behaviors of nurse managers have a profound impact on nursing team effectiveness, staff wellbeing, and quality of patient care (Zhou et al., 2024). In recent years, as healthcare environments grow increasingly complex and nursing workloads intensify, toxic leadership has emerged as a critical issue (Labrague, 2021; Özkan et al., 2022).

Toxic leadership is a negative style that usually manifests itself in the form of demeaning, jealous, authoritarian, intimidating, and manipulative subordinates, which can be harmful to employees and organizations (The Lancet, 2019; Yang et al., 2020). Labrague et al. (2020) categorized toxic leadership behaviors of nurse managers into intemperate behavior, narcissistic behavior, self-promoting behavior, and humiliating behavior. A qualitative study (Guo et al., 2022) further revealed specific manifestations such as negative feedback, neglecting subordinates, unfair treatment, self-centeredness, excessive pressure, and inaction. These behaviors may negatively impact nursing teams in multiple ways, including reduced job satisfaction, heightened professional burnout, increased turnover intentions, and nursing human resource attrition (Ofei et al., 2023). They may also foster organizational silence and deteriorate departmental morale (Hossny et al., 2023). According to a systematic review (Labrague, 2023), toxic leadership can trigger a chain of detrimental effects. It heightens nurses' organizational alienation, impairs their mental health, and diminishes work efficiency, ultimately jeopardizing patient clinical outcomes. Therefore, toxic leadership behaviors are not only detrimental to the psychological health of nurses, but also pose a threat to the quality of service and patient safety in healthcare organizations.

In the field of nursing management, research on leadership behaviors has undergone a more pronounced evolution. Early nursing leadership research centered on positive models like transformational and servant leadership, highlighting how leaders enhance team performance through motivation and personalized care (Wang et al., 2019; Yang et al., 2019). However, with the paradigm shift from positive to negative behavioral research, scholars have begun to focus on the phenomenon of negative leadership in the workplace. The high-intensity and emotionally charged nature of nursing may lead managers to adopt aggressive measures, underscoring the necessity of this research shift.

Currently, most of the research on toxic leadership behavior is concentrated in Western countries, focusing on its formation mechanisms and negative impacts. Estes (2013) surveyed 6,500 nurses in the United States found that 46.6% indicated that they had experienced toxic leadership behaviors by nurse leaders. Results of a survey of 943 nurses from a multi-center in Ghana revealed that nurses perceived toxic leadership behaviors of nurse managers at a moderate level (Ofei et al., 2022). In addition, several studies have found that nurse managers' toxic leadership behaviors can reduce nurses' work engagement and organizational performance levels, and increase nurses' propensity to leave and job stress (Semedo et al., 2022; Ahmed et al., 2024). There are fewer studies on toxic leadership behaviors in China. Zheng et al. (2024) investigated 1,310 nurses and concluded that toxic leadership behaviors of nurse managers can have a negative impact on clinical nurses' work engagement. Another survey (Yao et al., 2023) showed that 43.71% of nurses believed that nurse managers had mild toxic leadership behavior, suggesting that some nurses in China also faced the status quo of toxic leadership behavior.

The toxic leadership behaviors of nurse managers scale (ToxBH-NM) has been applied across regions, and currently stands as the sole instrument (Labrague et al., 2020). Despite advances in toxic leadership research, most scholars treat it solely as a continuous variable, overlooking potential qualitative differences and subgroup characteristics. In addition, research on the heterogeneity of exposure to toxic leadership behaviors across different nursing populations remains severely inadequate. These limitations not only constrain our deep understanding of toxic leadership behaviors but also hinder the development of targeted intervention measures.

Latent Profile Analysis (LPA) is a person-centered approach that uses model fitting to categorize individuals into unobserved (latent) subgroups based on their responses to a set of observed variables (Yang et al., 2022). Traditional clustering methods rigidly partition samples and rely on metrics like scatter plots and contour coefficients for model selection, resulting in inconsistent standards. In contrast, LPA emphasizes membership probabilities, transcending simple data grouping. It provides fit indices based on statistical tests to select appropriate models (Liu et al., 2022). This ultimately achieves grouping effects characterized by intergroup heterogeneity and intragroup homogeneity. It also exhibits high correlation with the precise identification of subgroup characteristics within distinct feature groups. This method aids in revealing qualitative differences and distribution patterns of toxic leadership behaviors within nursing contexts. It also enables in-depth analysis of the “targeted” patterns of toxic leadership behaviors and their underlying formation mechanisms.

In summary, exploring the group heterogeneity of toxic leadership behaviors among nursing managers holds significant value. Therefore, this study aimed to identify subgroups of toxic leadership behaviors among nursing managers, and investigate their influencing factors.

## Methods

### Design

An online survey was employed in this cross-sectional study to collect relevant data from the target population. Convenience sampling was utilized as the sampling method, aiming to enhance the feasibility and efficiency of the research by allowing access to readily available participants under limited resources. This study was designed and reported in accordance with the guidelines for Strengthening the Reporting of Observational Studies in Epidemiology (von Elm et al., 2014).

### Sample size estimation

Latent profile analysis (LPA) requires a sample size of 500 cases, therefore the minimum sample size for this study was 500 (Kassab et al., 2023).

### Participants

From May to December 2024, nurses in Guiyang City and Zunyi City, Guizhou Province were invited to conduct the survey. Inclusion criteria: age ≥ 18 years; ≥1 year in nursing; full-time registered nurses; informed consent to this survey and signed an informed consent form. Exclusion criteria: further training, regulation training or internship nurses.

### Measurements

#### Demographic and sociological information

A general information questionnaire was developed by the researchers, including basic demographic data such as age, gender, education level, professional title, employment types, nursing experience, hospital types, and department.

#### Toxic leadership behaviors of nurse managers scale (ToxBH-NM)

The scale was developed by Labrague et al. (2020). This study used the Chinese version translated by Yao et al. (2023), which was tested for reliability and validity and culturally debugged in China, and has good reliability and validity. The scale includes four dimensions of intemperate behavior, narcissistic behavior, self-promoting behavior, and humiliating behavior. This scale consists a total of 30 items. For the Chinese version of the scale, the Cronbach's α, split-half reliability, and test-retest reliability coefficients were 0.951, 0.831, and 0.959, respectively. The total score ranges from 30 to 150 points, with higher scores indicating more severe toxic leadership behaviors.

### Data collection

Wenjuanxing (www.wjx.cn) was used to develop a web-based questionnaire and two researchers reviewed the online questionnaire for rigor. We also created a poster to display the link and QR code, on which the inclusion and exclusion of the population in this study was clearly indicated. We distributed the questionnaire within Guiyang City and Zunyi City. The researcher contacted general nurses to distribute the questionnaires. WeChat was used to send posters and information letters. Participants could access and complete the survey by clicking a link or scanning a QR code. Once submitted, their responses were directly returned to the web page. After the data collection period, two researchers manually reviewed all responses to exclude invalid questionnaires.

### Statistical analyses

Mplus 8.3 software was used for LPA to test the model fitness by gradually increasing the profiles based on the fit index. The following fit indices were employed to evaluate the models: Akaike Information Criterion (AIC), Bayesian Information Criterion (BIC), and adjusted BIC (aBIC). For these indices, smaller values indicate a better model fit. Additionally, entropy was used to assess classification accuracy. It ranges from 0 to 1, with values closer to 1 representing higher accuracy. Lo-mendell-rubin likelihood ratio test (LMRT) and Bootstrapped likeli-hood ratio test (BLRT) are used to evaluate model fit, with a significant *P*-value (*P* < 0.05) suggesting that the k-class model fits better than the (k-1)-class model. Although these evaluation indices provide a reference for profile decision-making, the interpretability and theoretical justification of each profile should also be considered when determining the best model.

Data were statistically analyzed using SPSS29.0 software. Count data were expressed as frequencies and percentages or percentages, and comparisons between groups were made using chi-square test or rank-sum test. A multiple logistic regression was performed with the LPA classification as the dependent variable and the variables significant in univariate analysis as independent variables. The significance threshold was set at α = 0.05.

### Ethics considerations

The Ethics Committee of the Third Affiliated Hospital of Zunyi Medical University approved this study (2024-1-746). Informed consent was obtained from all participants after disclosure of the study's purpose. Participation was voluntary, with immediate termination of data collection upon refusal. Anonymity is guaranteed through aggregated reporting of results.

## Results

### Common method bias test

Since the research data is self-reported, common method bias may exist. During the survey process, we emphasized the anonymity and confidentiality of the questionnaire. We explicitly stated that the data would be used solely for academic research to minimize sources of common method bias. Additionally, the Harman single-factor test was employed to assess common method bias. Results revealed three unrotated factor loadings exceeding 1. The first factor explained 33.382% of variance (<40%), indicating that common method bias does not constitute a significant issue.

### Demographic characteristics

A total of 900 nurses were invited to participate in the survey, 9 refused, 15 filled out incorrectly, 8 filled out incomplete. Finally, 868 valid questionnaires were recovered, with an effective recovery rate of 96.44%. The ages of the 868 nurses were categorized into four groups: 18–25 (*n* = 58), 26–35 (*n* = 633), 36–45 (*n* = 154), and ≥46 (*n* = 23). Of the participants, 51 (5.9%) were male and 817 (94.1%) were female.

### Model fit indices of LPA

A model-fitting analysis was conducted to estimate profiles of nurses' toxic leadership, based on the dimension scores of the Chinese version of the ToxBH-NM. In this study, the model was fitted and estimated from an initial latent profile model, and then the number of profiles was gradually increased to determine the optimal model. Table 1 shows the fit indices of the latent profile models with different numbers of profiles. As the number of profiles increases, the values of AIC, BIC and aBIC decrease, indicating that the latent profile model is becoming more optimized. The Class 4 model demonstrated the highest classification accuracy, with a maximum entropy value of 0.925. Furthermore, the number of nurses in each of the four classes was 478, 145, 116, and 129, all of which exceeded the recommended threshold of 5% of the total sample size. Based on a comprehensive evaluation of the model fit indices, theoretical justification, and practical interpretability, the Class 4 model was selected as the optimal solution. To test the sensitivity of the results, a separate LPA was conducted on the female subgroup. The results also supported the previously identified Class 4 model (see Table A1). Consequently, the Class 4 model was selected as the final model for this study.

**Table 1:** Fit statistics for profile structure (*n* = 868).

**Model**	**AIC**	**BIC**	**aBIC**	**Entropy**	**LMRT (*P*)**	**BLRT (*P*)**	**Category probability**
1	22778.316	22816.445	22791.039	–	–	–	1
2	21711.942	21773.902	21732.618	0.920	<0.001	<0.001	0.692/0.308
3	21497.841	21583.632	21526.469	0.828	0.0001	<0.001	0.215/0.522/0.263
4	21174.237	21283.860	21210.817	0.925	<0.001	<0.001	0.551/0.134/0.167/0.149
5	21105.415	21238.868	21149.947	0.861	0.0156	<0.001	0.454/0.122/0.134/0.132/0.158

AIC, Akaike Information Criterion; BIC, Bayesian Information Criterion; aBIC, adjusted BIC; LMRT, Lo Mendell-Ruben adjusted likelihood ratio test; BLRT, bootstrap likelihood ratio test.

### The scale and dimension scores of different profiles

**Figure 1:**
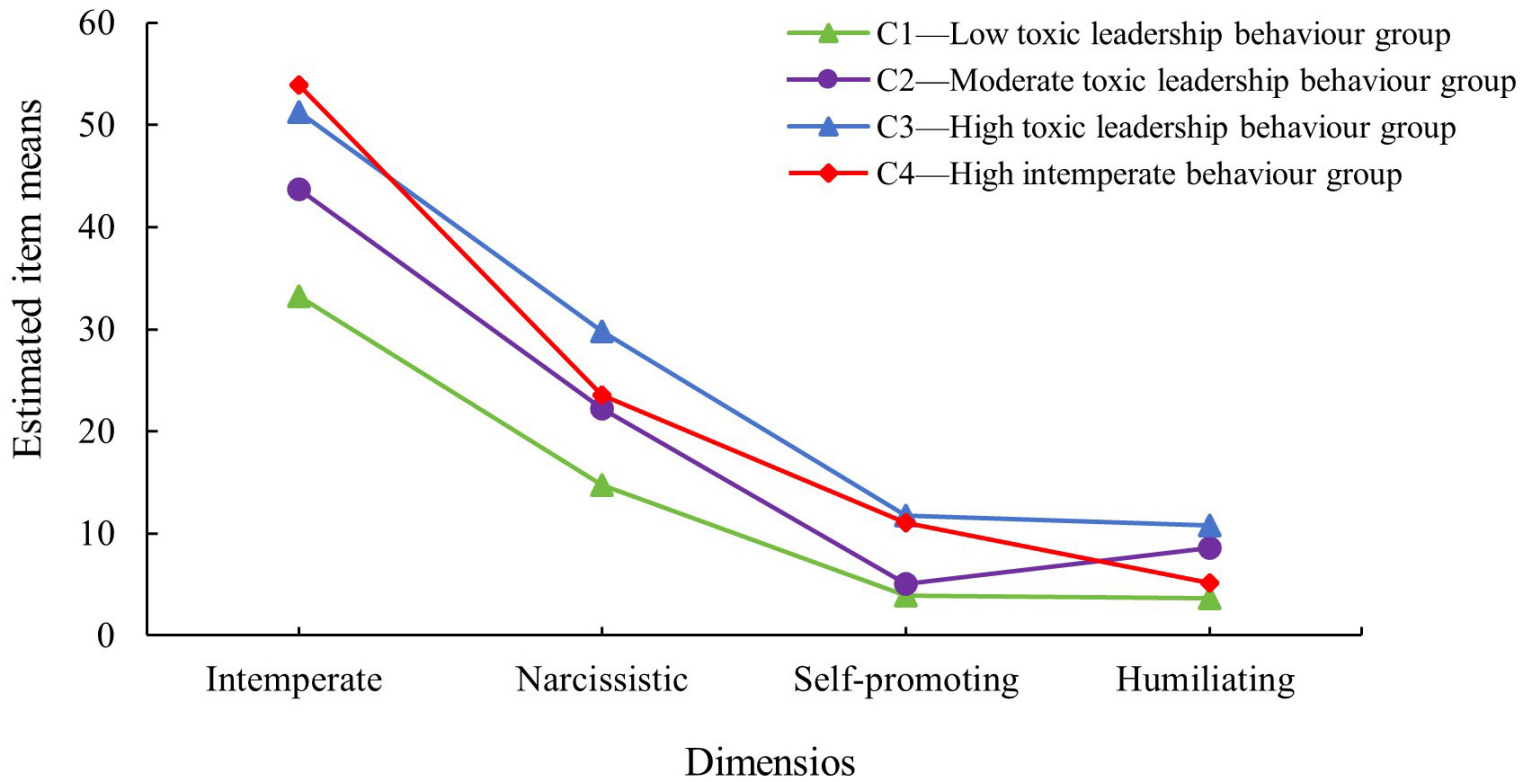
Latent profiles of toxic leadership behaviors of nurse managers.

Based on the LPA results, this study identified four distinct profiles of nurses' exposure to toxic leadership behaviors by nurse managers. Profile 1 (C1) was labeled the “Low Toxic Leadership Behavior Group.” Nurses in this profile scored low across all dimensions of toxic leadership, with a total score of 50 (42, 68). This group comprised 478 nurses, accounting for 55.07% of the total sample. Profile 2 (C2) was categorized as the “Moderate Toxic Leadership Behavior Group,” with a total score of 80 (61, 96). It included 145 nurses, representing 16.71% of the sample. Profile 3 (C3) was identified as the “High Toxic Leadership Behavior Group,” exhibiting a high total score of 106 (95, 116). This group consisted of 116 nurses, making up 13.36% of the sample. Profile 4 (C4) was termed the “High Intemperate Behavior Group,” characterized by high scores specifically on the Intemperate Behavior dimension and low scores on all other dimensions. It contained 129 nurses, accounting for 14.86% of the total sample. The LPA results is presented in Figure 1.

### Demographic and related characteristics of each profile

Following the identification of distinct nurse profiles via LPA, chi-square tests were used to assess for significant differences in demographic characteristics across the profiles. The results revealed statistically significant differences in gender, employment type, hospital type, and department across the profiles. The remaining variables did not show significant associations. The results are presented in the Table 2.

**Table 2:** Demographic and related characteristics of each profile (*n* = 868).

**Variables**	**Lowly toxic leadership behavior group (*n* = 478)**	**Moderately toxic leadership behavior group (*n* = 145)**	**Highly toxic leadership behavior group (*n* = 116)**	**Highly intemperate behavior group (*n* = 129)**	**χ^2^**	***P* value**
Age					6.524	0.687
18–25	32 (6.69)	8 (5.52)	13 (11.21)	5 (3.88)		
26–35	345 (72.18)	109 (75.17)	82 (70.69)	97 (75.19)		
36–45	88 (18.41)	24 (16.55)	19 (16.38)	23 (17.83)		
46~	13 (2.72)	4 (2.76)	2 (1.72)	4 (3.10)		
Gender					12.608	0.006
Male	17 (3.56)	14 (9.66)	12 (10.34)	8 (6.20)		
Female	461 (96.44)	131 (90.34)	104 (89.66)	121 (93.80)		
Education					1.192	0.755
College diploma	94 (19.67)	28 (19.31)	26 (22.41)	30 (23.26)		
Bachelor's degree and above	384 (80.33)	117 (80.69)	90 (77.59)	99 (76.74)		
Professional title					11.898	0.064
Junior-level	304 (63.60)	87 (60.00)	75 (64.66)	77 (59.69)		
Intermediate-level	164 (34.31)	48 (33.10)	39 (33.62)	44 (34.11)		
Senior-level	10 (2.09)	10 (6.90)	2 (1.72)	8 (6.20)		
Employment types					27.928	<0.001
Non-permanent staff	319 (66.74)	114 (78.62)	104 (89.66)	94 (72.87)		
Permanent employee	159 (33.26)	31 (21.38)	12 (10.34)	35 (27.13)		
Nursing experience (years)					19.046	0.087
<5	87 (18.20)	26 (17.93)	15 (12.93)	27 (20.93)		
5–10	196 (41.00)	73 (50.34)	51 (43.97)	52 (40.31)		
10–15	151 (31.59)	37 (25.52)	40 (34.48)	32 (24.81)		
15–20	27 (5.65)	1 (0.69)	6 (5.17)	8 (6.20)		
20~	17 (3.56)	8 (5.52)	4 (3.45)	10 (7.75)		
Hospital types					44.789	<0.001
General hospital	367 (76.78)	136 (93.79)	106 (91.38)	122 (94.57)		
Specialty hospital	111 (23.22)	9 (6.21)	10 (8.62)	7 (5.43)		
Department					51.264	<0.001
Internal medicine system	133 (27.82)	57 (39.31)	31 (26.72)	26 (20.16)		
Surgical system	131 (27.41)	29 (20.00)	41 (35.34)	33 (25.58)		
Gynecology and obstetrics system	45 (9.41)	10 (6.90)	10 (8.62)	16 (12.40)		
Pediatric system	41 (8.58)	17 (11.72)	9 (7.76)	20 (15.50)		
Outpatient and emergency system	38 (7.95)	15 (10.34)	18 (15.52)	10 (7.75)		
Critical care system	33 (6.90)	5 (3.45)	2 (1.72)	15 (11.63)		
Nursing department	38 (7.95)	6 (4.14)	2 (1.72)	4 (3.10)		
Function department	19 (3.97)	6 (4.14)	3 (2.59)	5 (3.88)		

### Results of multiple logistic regression analysis

Multiple logistic regression was performed using the four profiles as dependent variables, incorporating the significant univariate predictors of gender, employment type, hospital type, and department. Using C1 as the reference group, the results indicated that nurses who were male, employed as non-permanent staff, and working in general hospitals were more likely to be classified into C2. Similarly, male nurses and those working in general hospitals were more likely to belong to C3. Furthermore, nurses in general hospitals were also more likely to be categorized into C4. The results are presented in the Table 3.

**Table 3:** Results of multiple logistic regression analysis.

**Variables**	**Class 2 vs. class 1**			**Class 3 vs. class 1**			**Class 4 vs. class 1**		
	**β**	***OR* (95% CI)**	***P***	**β**	***OR* (95% CI)**	***P***	**β**	***OR* (95% CI)**	***P***
Gender (ref: female)	1.004	2.729 (1.211, 6.148)	0.015	0.892	2.440 (1.129, 5.277)	0.023	0.510	1.665 (0.679, 4.085)	0.266
Male									
Employment types (ref: permanent staff)	1.448	4.235 (2.247, 8.050)	<0.001	0.638	1.893 (1.2, 2.986)	0.006	0.317	1.374 (0.877, 2.150)	0.165
Non-permanent staff									
Hospital types (ref: specialty hospital)	1.324	3.759 (1.828, 7.730)	<0.001	1.608	4.993 (2.394, 10.414)	<0.001	1.832	6.246 (2.718, 14.355)	<0.001
General hospital									
Department (ref: function department)	0.213	1.237 (0.333, 4.599)	0.751	0.126	1.134 (0.416, 3.09)	0.805	−0.470	0.625 (0.209, 1.873)	0.402
Internal medicine system	0.562	1.755 (0.477, 6.451)	0.397	−0.479	0.620 (0.22, 1.743)	0.364	−0.175	0.840 (0.284, 2.481)	0.752
Surgical system	0.625	1.868 (0.442, 7.904)	0.396	−0.013	0.987 (0.3, 3.245)	0.983	0.651	1.917 (0.587, 6.265)	0.281
Gynecology and obstetrics system	0.061	1.063 (0.249, 4.542)	0.935	0.017	1.017 (0.334, 3.097)	0.976	0.360	1.433 (0.454, 4.526)	0.539
Pediatric system	0.830	2.293 (0.577, 9.118)	0.239	−0.055	0.946 (0.306, 2.927)	0.924	−0.283	0.753 (0.22, 2.582)	0.652
Outpatient and emergency system	−1.350	0.259 (0.038, 1.755)	0.167	−1.139	0.320 (0.083, 1.232)	0.098	0.165	1.180 (0.36, 3.865)	0.785
Critical care system	−0.832	0.435 (0.065, 2.933)	0.393	−0.548	0.578 (0.158, 2.113)	0.407	−0.848	0.428 (0.1, 1.833)	0.253

Ref, reference.

## Discussion

The purpose of this study was to determine the characteristics of toxic leadership behaviors of nurse managers. In this study, we surveyed 868 nurses. The results identified four subgroups, low toxic leadership behavior group, moderate toxic leadership behavior group, high toxic leadership behavior group, and high intemperate behavior group. Our study showed that most of the nurse managers had moderate or low toxic leadership behavior, which is consistent with the results of most studies (Ofei et al., 2022; Yao et al., 2023; Nonehkaran et al., 2023). It is suggested that there is significant heterogeneity in the toxic leadership behaviors suffered by nurses. This heterogeneity may be closely related to nurses' personal characteristics, work environment, and leader characteristics, among others (Labrague et al., 2021; Guo et al., 2023; Farghaly Abdelaliem and Abou Zeid, 2023).

Our study found that male nurses are at higher risk of experiencing toxic leadership behaviors. This finding is highly consistent with Gender Role Theory (GRT; Li et al., 2021). Nursing is often viewed as a “female” profession characterized by empathy and collaboration, which can place male nurses at risk of role conflict for challenging gender norms (Yu et al., 2025). This conflict can be compounded by managerial bias, as some managers may attribute male nurses' career choices to competence issues (Wong et al., 2025), resulting in stricter supervision or negative feedback, aligning with Beck's findings (Baker et al., 2023). In addition, in gender imbalanced workplaces, men are more likely to be “targeted” and their behavioral biases may be amplified (Guy et al., 2022). This cognitive bias may lead to the accumulation of negative evaluations of male nurses by managers, which may eventually evolve into toxic behaviors. Concurrently, establishing male nursing unions or mutual support groups could promote fairness and inclusivity in the workplace environment (Manzi and Heilman, 2021; Morgenroth et al., 2020).

The findings suggest that non-permanent staff nurses are a group that suffers from toxic leadership behaviors. Non-permanent staff nurses lack stable contracts, benefits, and career paths, leading managers to view them as easily replaceable (Galbany-Estragués et al., 2022). This power imbalance can encourage exploitative behaviors. Moreover, Conservation of Resources theory (Bakker and Demerouti, 2024), suggests that these nurses' limited bargaining power forces them to accept unreasonable workloads. The resulting high-pressure environment gradually erodes their willingness to resist, perpetuating a “silence-exploitation” loop (Majeed and Fatima, 2020). The high percentage of non-permanent employee nurses in this study validates this view. This suggests that a more inclusive and equitable nursing workplace ecosystem is still needed to address toxic leadership behaviors (Abdelrahman et al., 2025).

This study revealed that nurses in general hospitals were at significantly higher risk than nurses in specialized hospitals, especially in the group with a high incidence of uncontrolled behavior. This phenomenon stands in stark contrast to the safe, supportive, and empowering work environment advocated by the Magnet Hospital Concept (Yang et al., 2021). General hospitals have high patient flow, frequent examinations of various types, complex interpersonal relationships, and long decision-making chains. Managers under prolonged intense work pressure may resort to unconventional means to maintain work efficiency, which may lead to toxic behaviors, and Sexton's report confirms our findings (Sexton et al., 2022; Beaulieu et al., 2023). Therefore, leveraging the Magnet Hospital initiative's principles flat decision-making structures, open communication cultures, and substantive professional autonomy for nurses can foster a positive atmosphere (Abdelrahman et al., 2025; Harolds and Miller, 2022).

This study examined the relationship between other variables and toxic leadership behavior. First, the findings did not support the predictive role of age and educational attainment on toxic leadership behavior proposed by Guo et al. (2023), suggesting that the explanatory power of these demographic variables may vary across different contexts. Second, neither nurses' professional titles nor work experience significantly influenced toxic leadership behaviors. This indicates that individual-level professional credentials play a limited role in the formation of toxic leadership, suggesting its origins may stem more from organizational environments or leaders' inherent traits. Additionally, department affiliation did not demonstrate significant predictive power in this study, contradicting the findings of Guo et al. (2023). However, the observed distribution differences across departments suggest their potential influence should not be overlooked. These variations may stem from context-specific factors unique to each department, such as distinct work pressures, team cultures, or management styles. Consequently, further studies should aim to disentangle the precise mechanisms through which these factors interact, utilizing more detailed contextual analyses.

This study has some theoretical and practical implications for future research on toxic leadership behavior of nurse managers. First, nurses' gender, employment types and hospital types are the most significant factors affecting nurse managers' toxic leadership behaviors. Meanwhile, we should pay more attention to the degree of influence of these three factors and explore the mechanism of their effects from the theoretical level. Secondly, at the leadership level, hospitals must actively address toxic leadership behaviors faced by clinical nurses by implementing profile-specific countermeasures. This approach is essential for cultivating a healthy work atmosphere and boosting job satisfaction among nurses.

## Limitations

This study has several limitations. First, as the sample was recruited from a single province, and using convenience sampling results in a non-representative sample, the findings may lack generalizability to the broader national or global nursing population. Second, the cross-sectional design precludes the establishment of causal relationships. Finally, the use of self-reported data may introduce the potential for response biases.

## Conclusion

This study used LPA to identify subgroups of toxic leadership behaviors in nurse managers. The results showed that there were four subgroups: low toxic leadership behavior group, moderate toxic leadership behavior group, high toxic leadership behavior group, and high intemperate behavior group. This study suggests that hospital administrators should pay attention to the prevalence of toxic leadership behaviors among nurse managers. More importantly, targeted interventions based on the distinct profiles identified should be adopted, ultimately fostering a healthier work environment and improving nurse retention.

## Data Availability

The raw data supporting the conclusions of this article will be made available by the authors, without undue reservation.

## APPENDIX A

**Table A1:** Fit statistics for profile structure of female nurse.

**Model**	**AIC**	**BIC**	**aBIC**	**Entropy**	**LMR (*P*)**	**BLRT (*P*)**	**Category probability**
1	21396.651	21434.296	21408.891	–	–	–	1
2	21404.550	20475.723	20434.440	0.911	<0.001	<0.001	0.697/0.303
3	20193.517	20278.218	20221.057	0.833	0.0001	0.0002	0.221/0.526/0.253
4	19904.519	20012.749	19939.710	0.925	<0.001	<0.001	0.152/0.164/0.558/0.127
5	19831.251	19963.009	19874.092	0.864	0.0174	0.0197	0.464/0.125/0.131/0.145/0.136
